# Shielding resources for four common radiopharmaceuticals utilized for imaging and therapy: Tc‐99m, F‐18, I‐131, and Lu‐177

**DOI:** 10.1002/acm2.70084

**Published:** 2025-03-24

**Authors:** Michael Oumano, Richard Wendt, James Botti, Nathan Busse, David Hintenlang, Stephanie Leon, Kevin Little, Melissa Martin, Richard Massoth, Kenneth Matthews, Rameshwar Prasad, Sharon White, Jessica Clements

**Affiliations:** ^1^ Department of Medicine and Biological Sciences Brown University Providence Rhode Island USA; ^2^ Department of Medical Physics and Radiation Safety Rhode Island Hospital Providence Rhode Island USA; ^3^ Landauer Medical Physics Glenwood Illinois USA; ^4^ UT MD Anderson Cancer Center Houston Texas USA; ^5^ Nuclear Medicine Medical Physics Consultants Ann Arbor Michigan USA; ^6^ Colorado Associates in Medical Physics Denver Colorado USA; ^7^ Department of Radiology Ohio State University Columbus Ohio USA; ^8^ Department of Radiology University of Florida Gainesville Florida USA; ^9^ Therapy Physics Inc. Signal Hill California USA; ^10^ Sunflower Medical Physics LLC Sioux Falls South Dakota USA; ^11^ Department of Physics and Astronomy Louisiana State University Baton Rouge Louisiana USA; ^12^ Department of Radiation Oncology UT Southwestern Medical Center Dallas Texas USA; ^13^ Department of Radiology University of Alabama at Birmingham Birmingham Alabama USA; ^14^ University of Vermont Medical Center Burlington Vermont USA

**Keywords:** Lu‐177, nuclear medicine, radiation safety, shielding, theranostics

## Abstract

**Introduction:**

The use of radioactive materials in the United States has been tightly regulated by the Nuclear Regulatory Commission and other entities for many decades. In 2015, however, the Joint Commission began to require hospital‐based nuclear medicine departments to conduct shielding designs and evaluations for radioactive material areas, mirroring established x‐ray practices. NCRP Report No. 147 guides diagnostic medical x‐ray shielding, but obviously cannot be used alone for nuclear medicine applications. The rising demand for theranostic nuclear medicine shielding evaluations particularly necessitates updated focused guidance, the aim of this report.

**Methods:**

Monte Carlo simulations were conducted using GATE software to analyze the effects of various barriers on the transmission of radioactive emissions. The simulations used point sources of Tc‐99m, F‐18, I‐131, and Lu‐177 and evaluated dose deposition to blocks of tissue using Dose Actors. Different ceiling heights (ranging from 10–16 feet) were also tested for scattering effects. The Archer equation was employed to fit transmission curves and estimate required barrier thicknesses.

**Results:**

Broad beam transmission factors and Archer fitting parameters are reported for various materials including lead, gypsum, concrete (light weight and normal weight), glass, and steel. A sample shielding calculation is provided for a wall separating Lu‐177 dotatate patients from an adjacent office to maintain public dose limits. Relevant occupancy factors are also provided.

**Conclusions:**

While Lu‐177 has a relatively low exposure rate constant, shielding may be necessary for high‐volume therapies like Lu‐177 DOTATATE and Lu‐177 vipivotide tetraxetan PSMA. Shielding involves accounting for broad radiation beams and requires thorough characterization of radiation buildup. When shielding to the typical height of 7 feet, scatter from ceilings and floors is negligible for transmission above 10%, but severely limits the ability to shield for transmission below 1%.

## 1 INTRODUCTION

The use of radioactive material has been carefully regulated in the United States for many decades by the Nuclear Regulatory Commission (NRC), agreement states’ radiation control programs, and others. However, it was not until 2015 that the Joint Commission became the first organization to require hospital‐based nuclear medicine departments to begin performing shielding designs and evaluations for areas where radioactive materials are used or stored.^1^, ^2^ Shielding designs and post‐installation radiation protection surveys have long been a common practice with x‐ray producing equipment, and the Joint Commission's 2015 requirement has simply brought the same approach to nuclear medicine.

Because of the regulatory needs and the complicated nature of shielding ionizing radiation for x‐ray producing equipment, the National Council on Radiation Protection and Measurement (NCRP) released Report No. 147^3^ in 2004 to assist medical physicists and health physicists in x‐ray shielding‐related tasks. NCRP Report No. 147 is still used today as the primary guidance document on this subject and many rules and regulations explicitly require using its methodology when planning to use x‐ray producing equipment in medical diagnostic applications. However useful it is in an x‐ray context; this report cannot be used by itself in nuclear medicine applications. The characteristics of the radiations that are emitted by the radionuclides, shielding materials and clinical workflows that are used in nuclear medicine are substantially different from those that are associated with x‐ray sources that are discussed in NCRP Report No. 147, so there is a need for similar guidance that is focused on nuclear medicine applications.

The approach that is taken here parallels that of Task Group 108 (TG108) of the American Association of Physicists in Medicine, which published Report No. 108^4^ in 2006. That report is specific to positron emission tomography (PET) and PET/CT imaging. The four radionuclides that are discussed here, Tc‐99m, F‐18, I‐131, and Lu‐177, were chosen to encompass both the predominant radionuclides that are used in diagnostic nuclear medicine as well as those that account for most of the currently approved treatments in the field of relatively new field of theranostics. The authors anticipate future reports addressing additional radionuclides and their clinical applications.

Since 2015, theranostic nuclear medicine procedures have also been growing in number and variety. They have already produced a significant rise in the demand for Nuclear Medicine shielding evaluations.^5^, ^6^, ^7^, ^8^ The urgency of this topic has led the authors to prioritize providing guidance on shielding for these applications, focusing on isotopes that are most relevant to current nuclear medicine and addressing a pressing clinical need.

### 1.1 Narrow beam versus broad beam geometries and buildup factors

**FIGURE 1:**
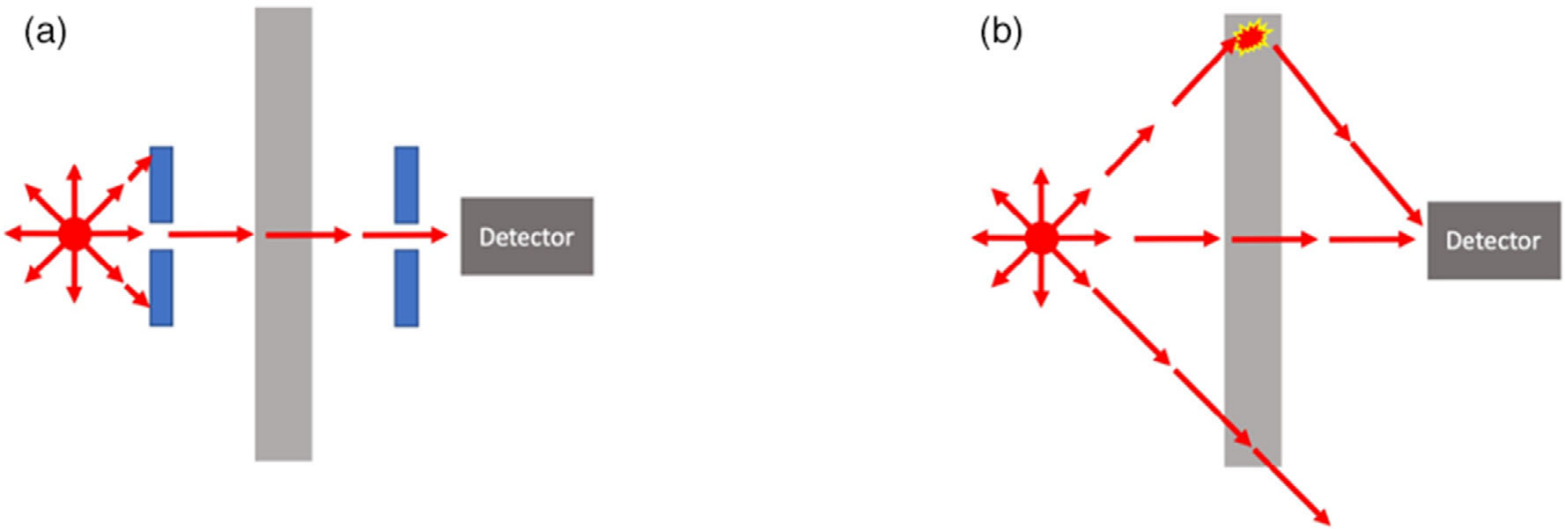
(a) Narrow beam geometry where scatter from outside the collimated path is negligible. (b) Broad beam geometry where photons are significantly scattered and redirected toward the detector. Note that this is a simple two‐dimensional depiction of what is in reality a three‐dimensional process.

When gamma rays and x‐rays are emitted toward a detector (or any point of concern) and collimated so that they cannot be significantly scattered from outside of the direct path toward the detector, a narrow beam geometry can be assumed, and the fraction of unattenuated photons that passes through the shield can be calculated by:

(1) $$Transmission=\frac{I}{I_{0}}=e^{-\mu *x}$$

where μ is the linear attenuation coefficient of the shielding material for the given photon's energy, *I* is the photon intensity incident upon the detector, *I_0_* is the initial photon intensity emitted toward the detector, and *x* is the shield's thickness. This scenario is depicted in Figure 1a.

However, the gamma rays and characteristic x‐rays that are emitted from patients in nuclear medicine who have been administered radioactive material are emitted in all directions and are uncollimated. Therefore, any detector or point of concern will also receive absorbed dose contributions from photons scattered into the detector by the shield itself, air, and other nearby materials as illustrated in Figure 1b.

Therefore, Equation 1 becomes Equation 2 in broad beam scenarios:

(2) $$Transmission=\frac{I}{I_{0}}=Be^{-\mu *x}$$

where *B* is the buildup factor with a value greater than unity. *B* depends on many factors such as the incident photon energy, the barrier's thickness, material composition, and shape, the presence of other surrounding material, the distance of the source to the barrier, and the distance of the detector to the barrier. This report develops broad beam transmission factors, which have buildup factors inherently included, that are analogous to the transmission factors in TG 108.

### 1.2 Lu‐177 therapies

While the use of Tc‐99m, F‐18, and I‐131 in Nuclear Medicine is well‐established, Lu‐177‐labeled compounds are relatively new in many nuclear medicine departments. A critical requirement when shielding for radiopharmaceuticals is defining the amount of time that the radionuclide will be located at a particular point in space. This will vary significantly for different nuclear medicine procedures. For example, Lu‐177 DOTATATE requires the administration of an amino acid solution (as well as antiemetics) before, during and after the slow infusion of the radiopharmaceutical. Patients are also often instructed to stay in the department for some period of time and to urinate frequently in order both to reduce their retained activity in non‐target tissues when they are discharged and for post‐therapy imaging. This means that the patient may spend about 2–4 h in the department after the administration of the standard dosage of 200 mCi of Lu‐177. This treatment is repeated every 8 weeks for up to four cycles per patient.

Similar to Lu‐177 DOTATATE, 200 mCi is the typical recommended dosage for Lu‐177 vipivotide tetraxetan PSMA. However, there are several key differences between the two therapies that are relevant to radiation shielding. In particular, there is no infusion of amino acids with Lu‐177 vipivotide tetraxetan PSMA so the amount of time that is, required for the patient to stay in the department for medical reasons is significantly shorter. We assume that these patients will have to spend as little as 30 min in the department for an administration, which is repeated every 6 weeks for as many as six cycles. Because Lu‐177 vipivotide tetraxetan PSMA clears from the body with a bi‐exponential time‐activity curve,^9^ some centers elect to hold the patients, some of whom are prone to urinary incontinence, for 2h after administration so that the patients can excrete more of the rapidly clearing component in a setting that is, under the control of the licensee. This is also desirable if post‐therapy imaging is to be performed on the same day.

## 2 MATERIALS AND METHODS

### 2.1 Monte Carlo models

**FIGURE 2:**
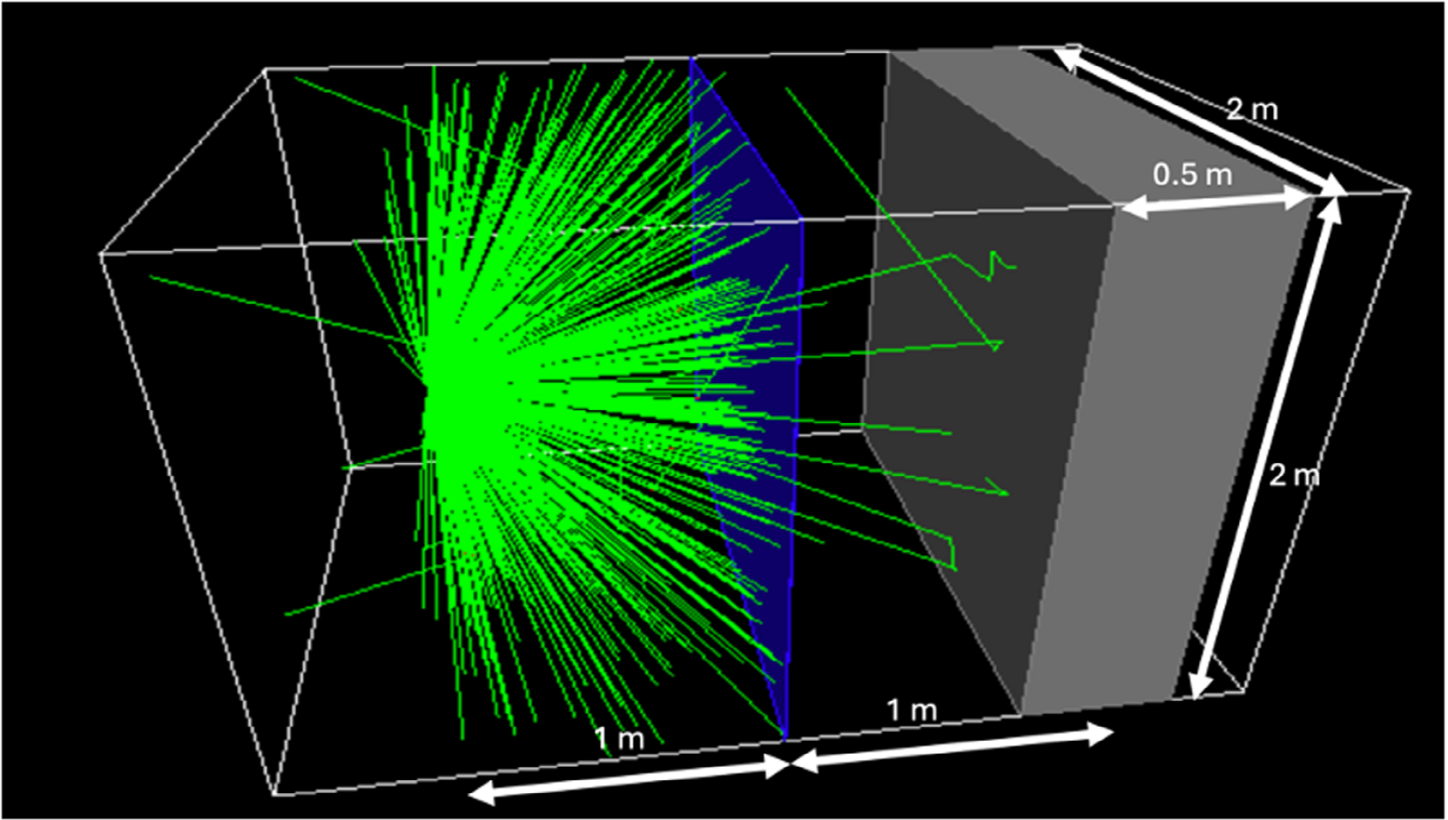
GATE Monte Carlo model of the source, the barrier, and the tissue target.

Monte Carlo simulations of the simple situation of the effect of a barrier of effectively infinite extent were independently run using GATE (version 9.2)^10^ and MCNP6 (MCNP version 6).^11^ In GATE, the simulation occurs within a so‐called universe that is, 3 m long, 2 m tall, and 2 m wide. It is filled with air. A 2m × 2m barrier is positioned with a normal to its surface parallel to the 3 m long axis of the universe so that it completely separates one side from the other. A characteristic of the simulations is that any particles that leave the universe simply disappear; there is no scattering from outside of the universe back into it. The barrier remains centered at that location although its thickness changes to simulate barriers of different thickness. A point source of the radionuclide is located at a distance of 1 m from the barrier and at a distance of 1 m from the bottom and top of the universe and at a distance of 1 m from the two sides of the universe. A block of tissue (modeled as muscle) that is 2 m tall and wide and 50 cm thick is located on the other side of the barrier from the point source. The 50 cm dimension is parallel to the 3 m dimension of the universe. The central plane of the tissue block is located at a distance of 1 m from the center of the barrier. Thus, there is a distance of 1.75 m between the point source and the proximal face of the tissue block. This is illustrated in Figure 2 below.

The source is constrained to emit in the 2π solid angle that is, oriented toward the barrier. This halves the simulation time while having a negligible effect on the results. The source is constrained to emit only the photons listed in ICRP107^12^ that exceed 15 keV in energy and 100 ppm in abundance. No charged particle emissions were included. These were the assumptions that were used by Smith and Stabin^13^ in their analysis of the shielding afforded by lead for many radionuclides.

Two billion emissions were simulated. A so‐called Dose Actor was attached to the tissue block. It records the absorbed dose that is deposited in each of its voxels, which were 5 mm in the direction parallel to the long axis of the universe and 2.5 mm in the directions perpendicular to the long axis. The average dose near the center of each plane of the tissue block was obtained by averaging the dose in each voxel within a region of interest (ROI) that was 150 mm in diameter over the center of the plane. Because the U.S. radiation protection regulations (e.g., 10CFR20.1201 and 10CFR20.1301) are stated in terms of deep dose equivalent (DDE) (e.g., 10CFR20.1003), the dose for each simulation was obtained by averaging the doses in the second and third 5 mm‐thick planes in order to estimate the simulated dose at a depth of 1 cm into the tissue block.

The doses that were reported by the Dose Actor were divided by the number of disintegrations that had been simulated so that the final data are in units of Gy/Bq‐s. The transmission factor of a particular barrier was then calculated by dividing the dose through that barrier by the dose through air. The definitions of the materials that were used in the simulations are given in Table 1. Various thicknesses of each barrier material were simulated for each of the radionuclides. In general, thicknesses up to those that result in a transmission factor of less than 0.001 were investigated. While gypsum is a common building material, in practical thicknesses it offers negligible attenuation of the emissions of F‐18 and I‐131 and thus was excluded as a significant barrier material for those two radionuclides.

**TABLE 1:** Materials used in GATE and MCNP6 Monte Carlo simulations.

Material	Reference	Density (g/cm^3^)	Elemental composition (mass fraction)
Air	14	0.001205	Nitrogen (0.755268), Oxygen (0.231781), Argon (0.012827), Carbon (0.000124)
Glass	10	2.5	Sodium (0.1020), Calcium (0.0510), Silicon (0.2480), Oxygen (0.5990)
Concrete	15	1.6 (LW) 2.3 (NW)	Hydrogen (0.01), Carbon (0.001), Oxygen (0.529107), Sodium (0.016), Magnesium (0.002), Aluminum (0.033872), Silicon (0.337021), Potassium (0.013), Calcium (0.044), Iron (0.014)
A514 Steel	16	7.85	Iron (0.97), Manganese (0.0095), Chromium (0.0065), Silicon (0.006), Molybdenum (0.0023), Carbon (0.004675), Zirconium (0.001), Boron (0.000025)
Gypsum	10	2.33	Hydrogen (0.0234), Oxygen (0.55757), Sulfur (0.186218), Calcium (0.23279)
Muscle	10	1.05	Hydrogen (0.102), Carbon (0.143), Nitrogen (0.034), Oxygen (0.71), Sodium (0.001), Phosphorus (0.002), Sulfur (0.003), Chlorine (0.001), Potassium (0.004)

Abbreviations: NW, Normal‐Weight; LW, Light‐Weight.

Similar simulations were run in MCNP6 as a secondary check to assure the authors the validity of the results calculated using GATE. The primary difference was that air kerma was simulated in MCNP6 using 5 cm‐radius spherical detectors made of air at different locations to test the effect of buildup as a function of distance. Six of these detectors were positioned between 30 cm and 180 cm from the wall at 30 cm increments. Individual simulations were also run for point sources at 1, 2, 3, 4, and 5 m from the wall on the opposite side. The F6 tally was used.

### 2.2 Scatter from the ceiling and floor

**FIGURE 3:**
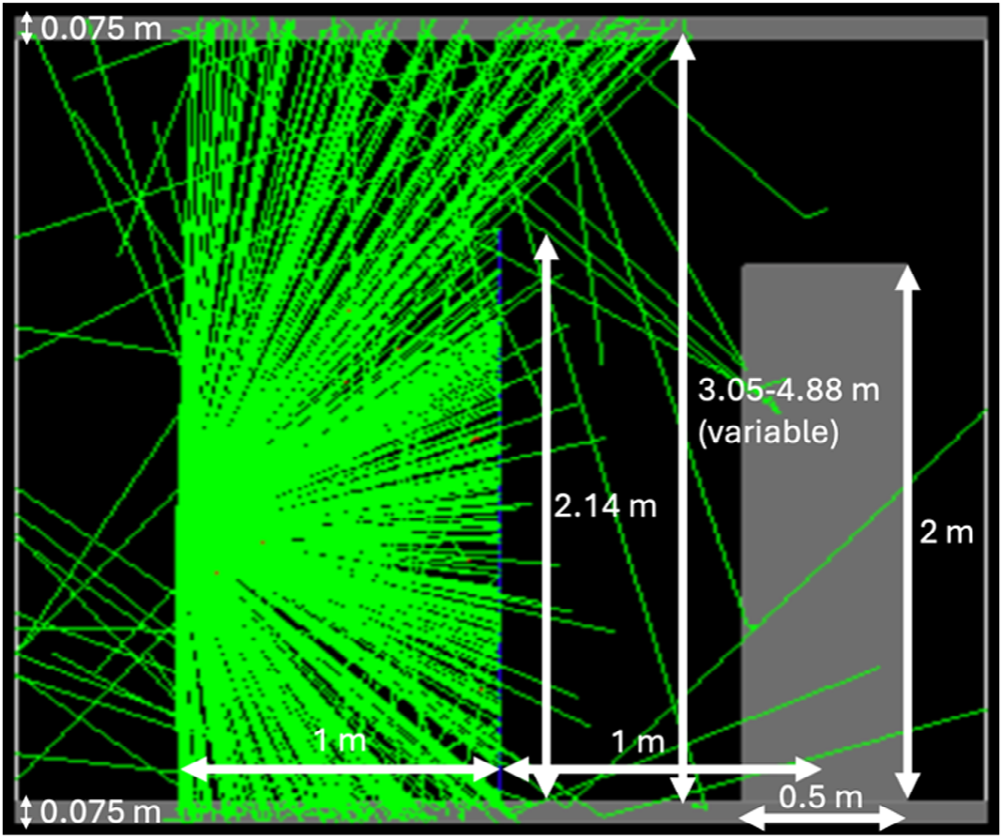
Monte Carlo geometry used in GATE to quantify scatter from the ceiling and floor.

Figure 3 depicts the model in GATE that was used to quantify the effect of scattering from the ceiling and floor. The target was again a block of tissue that was 2 × 2 m facing the source and 50 cm thick. It was resting on a floor of light weight concrete (see Table 1) that was 7.5 cm (3 in.) thick (i.e., its center was 1 m above the floor). There was a ceiling of light‐weight (LW) concrete that was also 7.5 cm (3 in.) thick. This is actually the floor above as distinct from a suspended ceiling that offers no appreciable attenuation. Simulations were run for the ceiling at heights of 3.05, 3.66, 4.27, and 4.88 m (10, 12, 14, and 16 feet respectively) from the floor in order to test the effect of ceiling height for a number of typical ceiling heights that have been encountered by the authors in facilities that are located in the United States. The barrier was 2.14 m (7 feet) wide and 2.14 m (7 feet) tall. Lastly, as the thickness of the barrier was varied, the dose ROI was positioned on the vertical face of the tissue block. However, as the transmission factor of the barrier dropped below 0.01 for more attenuating barriers, the position of the ROI was moved to the top face of the tissue block (closer to ceiling).

The effect of different distances between the source and the tissue block on the dose to the top of the tissue block from scatter was investigated by positioning the source at 1, 2, or 3 m from the center of a thick barrier and positioning the face of the tissue block 1, 2 or 3 m from the center of the barrier and recording the dose at a depth of 1 cm into the top of the tissue block in an ROI that was located 10 cm from the front edge of the tissue block.

### 2.3 Archer curve fitting

The transmission factors, which are based upon the estimated dose at a depth of 1 cm into the tissue block in the presence of a barrier divided by the dose at that depth in the absence of a barrier, were plotted as a function of the thickness of the barrier. These curves were fit to the Archer Equation^17^:

(3) $$Transmission={\left[\left(1+\frac{\beta }{\alpha }\right)e^{\alpha \gamma x}-\frac{\beta }{\alpha }\right]}^{-\left(\frac{1}{\gamma }\right)}$$

using the Prism software (version 9, Graphpad Software, San Diego, California) with the custom entry of the Archer functional form into Prism. In the case of Lu‐177 and lead, the parameters were uniquely determined by trial and error using a visual comparison of the fitting curve to the data points.

The Archer equation can be solved for the required barrier thickness, *x*, given the three parameters and the desired transmission factor, T:

(4) x=1αγlnT−γ+ββαα1+ββαα

## 3 RESULTS

### 3.1 Transmission factors and buildup

**FIGURE 4:**
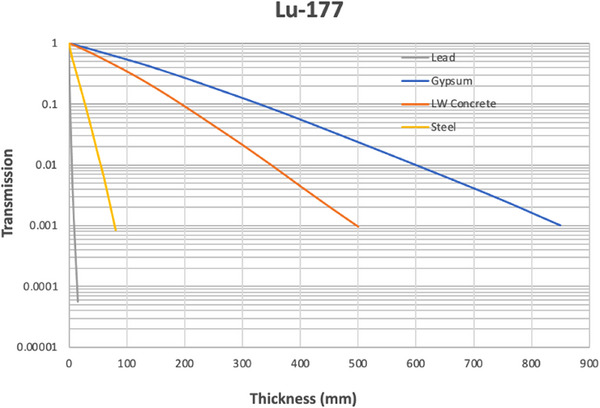
Broad beam transmission curves for Lu‐177.

**FIGURE 5:**
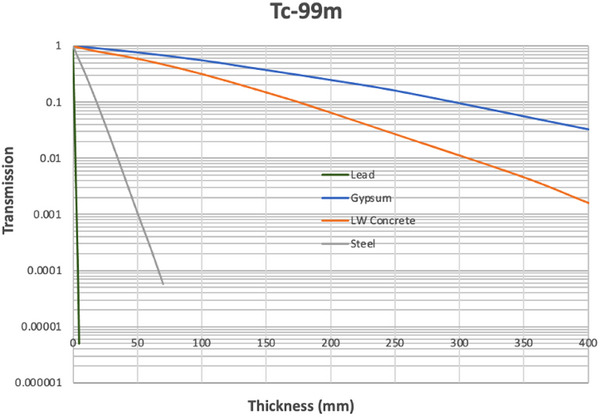
Broad beam transmission curves for Tc‐99m.

**FIGURE 6:**
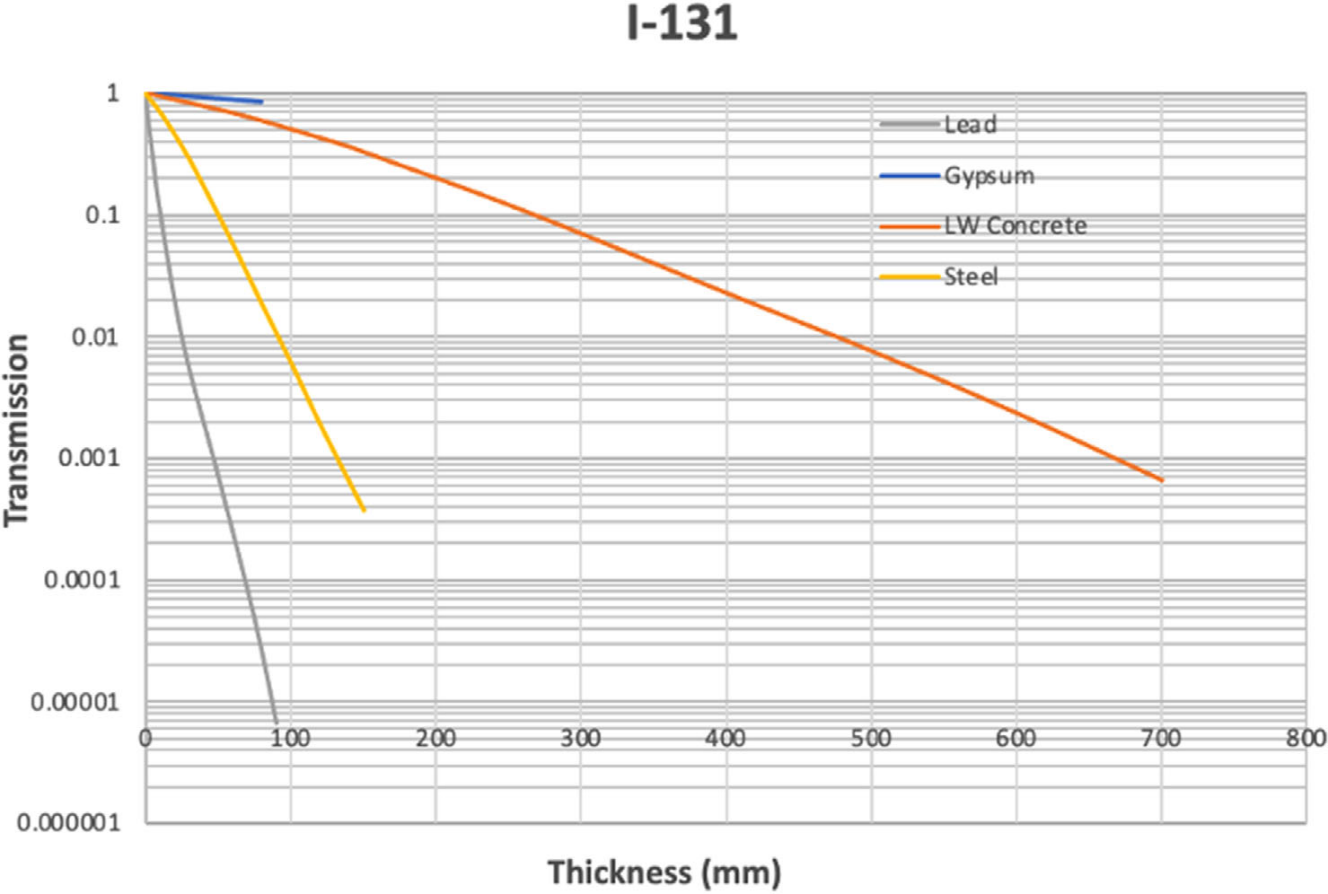
Broad beam transmission curves for I‐131.

Figures 4, 5, 6 show the results of the Monte Carlo simulations performed in GATE with the geometry depicted in Figure 2. There is generally very good agreement between the independent GATE and MCNP6 simulations. The differences become significant only once the transmission drops below about 0.01. We suspect that this is due to the dominance of scatter off the ceiling and floor at this point and a larger dependence on the positioning of sources and detectors.

**FIGURE 7:**
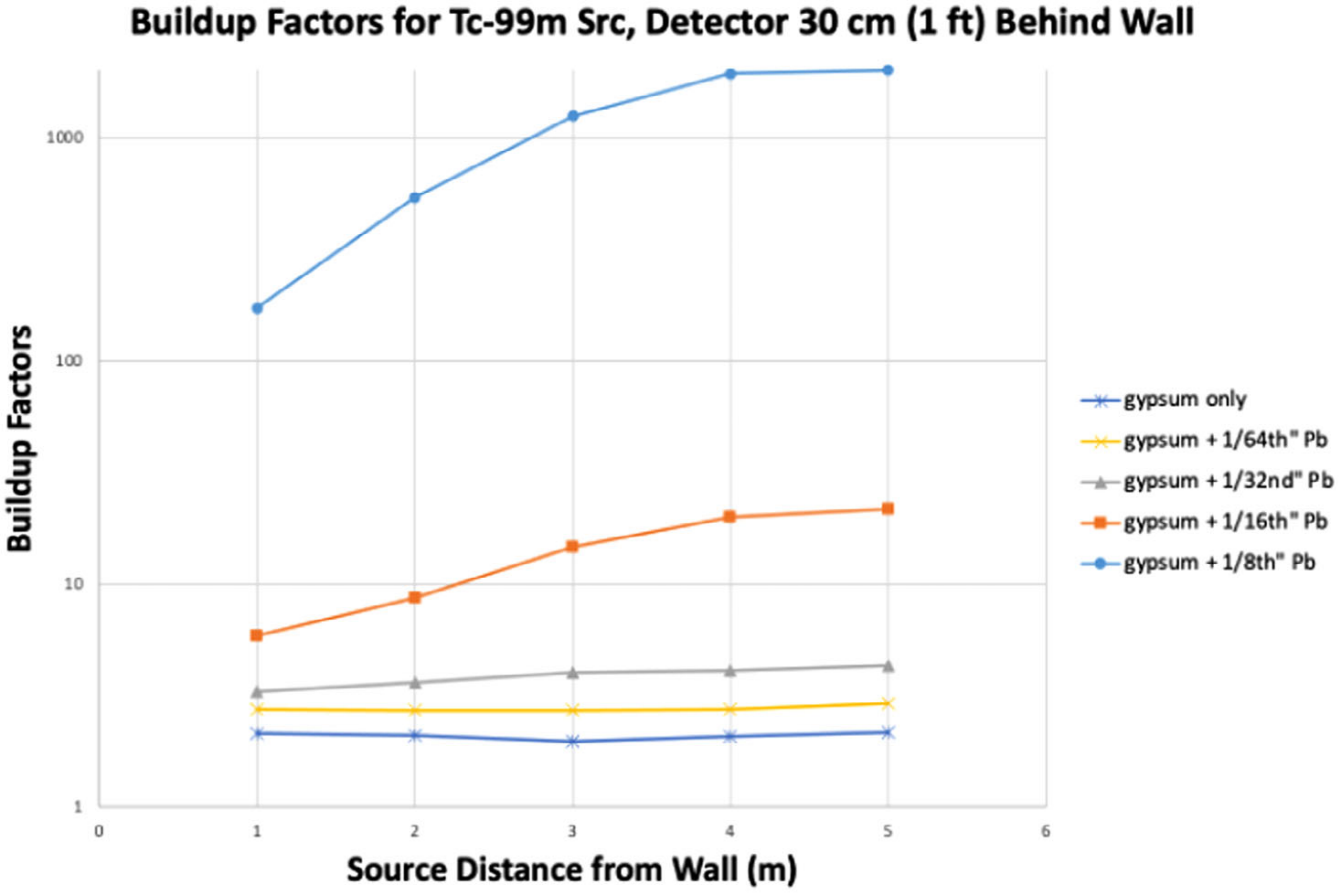
Buildup factors for Tc‐99m with the detector 1 ft behind the wall.

Figure 7 isolates the buildup factor, B, for Tc‐99m at various positions and thicknesses of lead plus gypsum wall board. It shows that B can range over multiple orders of magnitude (from about 2 to 2000) – mostly depending on the barrier thickness.

Table 2 gives fitting parameters for the Archer equation, Equation 3, for the four radionuclides and six barrier materials that are considered in this report. The parameters for F‐18 through lead from the TG108 report^4^ are included for comparison.

**TABLE 2:** Archer equation parameters (for thicknesses in mm) based upon the simulations using GATE.

Radionuclide	Barrier	α	β	γ
Tc‐99m	Lead	2.558	1.010	4.344
Tc‐99m	Gypsum	0.009549	−0.005312	1.430
Tc‐99m	LW concrete	0.02047	−0.01122	0.4389
Tc‐99m	NW concrete	0.03102	−0.01729	0.3622
Tc‐99m	514 Steel	0.1581	−0.04346	0.2602
Tc‐99m	Glass	0.03419	−0.02009	0.3076
Lu‐177	Lead	0.3855	1.071	0.2822
Lu‐177	Gypsum	0.009594	−0.003783	0.3739
Lu‐177	LW concrete	0.01615	−0.007056	0.5194
Lu‐177	NW concrete	0.02477	−0.01173	0.4404
Lu‐177	514 Steel	0.0797	2.243	28.74
Lu‐177	Glass	0.02456	−0.01197	0.6480
I‐131	Lead	0.1082	0.2072	0.5385
I‐131	LW concrete	0.01363	−0.007896	0.4847
I‐131	NW concrete	0.02062	−0.01220	0.4179
I‐131	514 Steel	0.05786	−0.02574	0.8742
I‐131	Glass	0.02191	−0.01319	0.4497
F‐18	Lead	0.166	−0.02184	0.2436
F‐18 TG‐108	Lead	0.1543	−0.04406	2.133
F‐18	LW concrete	0.01126	−0.006463	0.7475
F‐18	NW concrete	0.01558	−0.008775	0.8600
F‐18 TG‐108	NW concrete	0.01539	−0.01161	2.076
F‐18	514 Steel	0.05032	−0.02632	1.223
F‐18 TG‐108	Iron	0.05705	−0.03063	0.6319

Abbreviations:NW, noraml‐weight; LW, light‐weight.

Table 3 gives the fractional value layers that are calculated for the Archer equation parameters above using Equation 4, for the half‐value layer (HVL, T = 0.5), quarter‐value layer (QVL, T = 0.25), tenth‐value layer (TVL, T = 0.1), hundredth‐value layer (CVL, T = 0.01) and thousandth‐value layer (MVL, T = 0.001). The table also includes the fractional value layer thicknesses of lead for these four radionuclides from the work by Smith and Stabin,^13^ denoted by “S&S”, for comparison.

**TABLE 3:** Fractional value layer thicknesses (mm) calculated from the inverse Archer equation and also for lead barriers from Smith and Stabin^13^ ^.^

Radionuclide	Barrier	HVL (T = 0.5)	QVL (T = 0.25)	TVL (T = 0.1)	CVL (T = 0.01)	MVL (T = 0.001)
Tc‐99m	Lead	0.243	0.512	0.870	1.77	2.67
Tc‐99m S&S	Lead	0.234	0.535	0.905	1.8	2.7
Tc‐99m	Gypsum	115	199	299	542	783
Tc‐99m	LW concrete	64.6	117	176	305	423
Tc‐99m	NW concrete	44.3	80.6	122	211	291
Tc‐99m	514 Steel	5.86	11.4	18.4	34.8	50.4
Tc‐99m	Glass	43.3	78.8	119	204	279
Lu‐177	Lead	0.511	1.10	1.99	4.91	8.76
Lu‐177 S&S	Lead	0.542	1.19	2.11	4.7	8.46
Lu‐177	Gypsum	111	210	329	599	851
Lu‐177	LW concrete	68.0	126	194	349	495
Lu‐177	NW concrete	47.5	87.5	134	239	336
Lu‐177	514 Steel	7.23	15.9	27.4	56.3	85.2
Lu‐177	Glass	46.8	84.5	128	228	323
I‐131	Lead	2.48	5.54	10.5	26.8	46.3
I‐131 S&S	Lead	2.74	5.59	9.93	25.9	45.3
I‐131	LW concrete	101	180	268	459	635
I‐131	NW concrete	69.7	124	186	317	435
I‐131	514 Steel	18.1	32.8	50.2	91.1	131
I‐131	Glass	66.2	117	174	296	406
F‐18	Lead	4.75	9.40	15.4	30.1	44.5
F‐18 S&S	Lead	4.95	9.46	15.1	28.9	42.5
F‐18	LW concrete	113	197	293	508	714
F‐18	NW concrete	78.6	137	204	357	505
F‐18	514 Steel	21.7	37.9	57.3	104	149

Abbreviations: HVL, half‐value layer; QVL, quater‐value layer; TVL, tenth‐value‐layer; CVL, hundredth‐value layer; MVL, thousandth‐value layer; NW, normal‐weight LW, light‐weight.

### 3.2 Scatter off ceilings and floors

**FIGURE 8:**
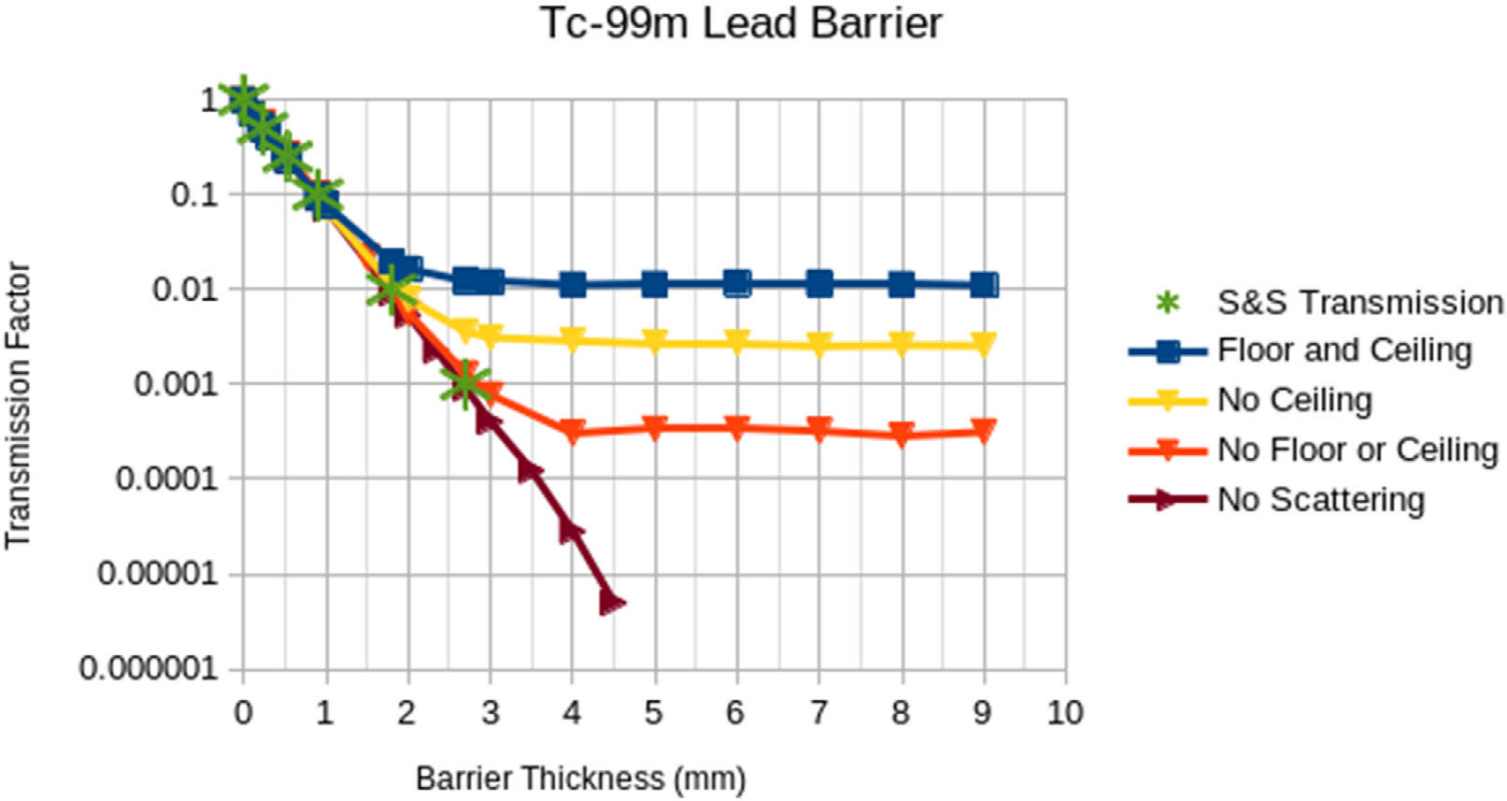
Broad beam transmissions curves for Tc‐99m and lead with and without scatter off ceiling and floor. Note that “no scattering” refers to a scenario with a barrier that extends to the edges of the simulation.

**FIGURE 9:**
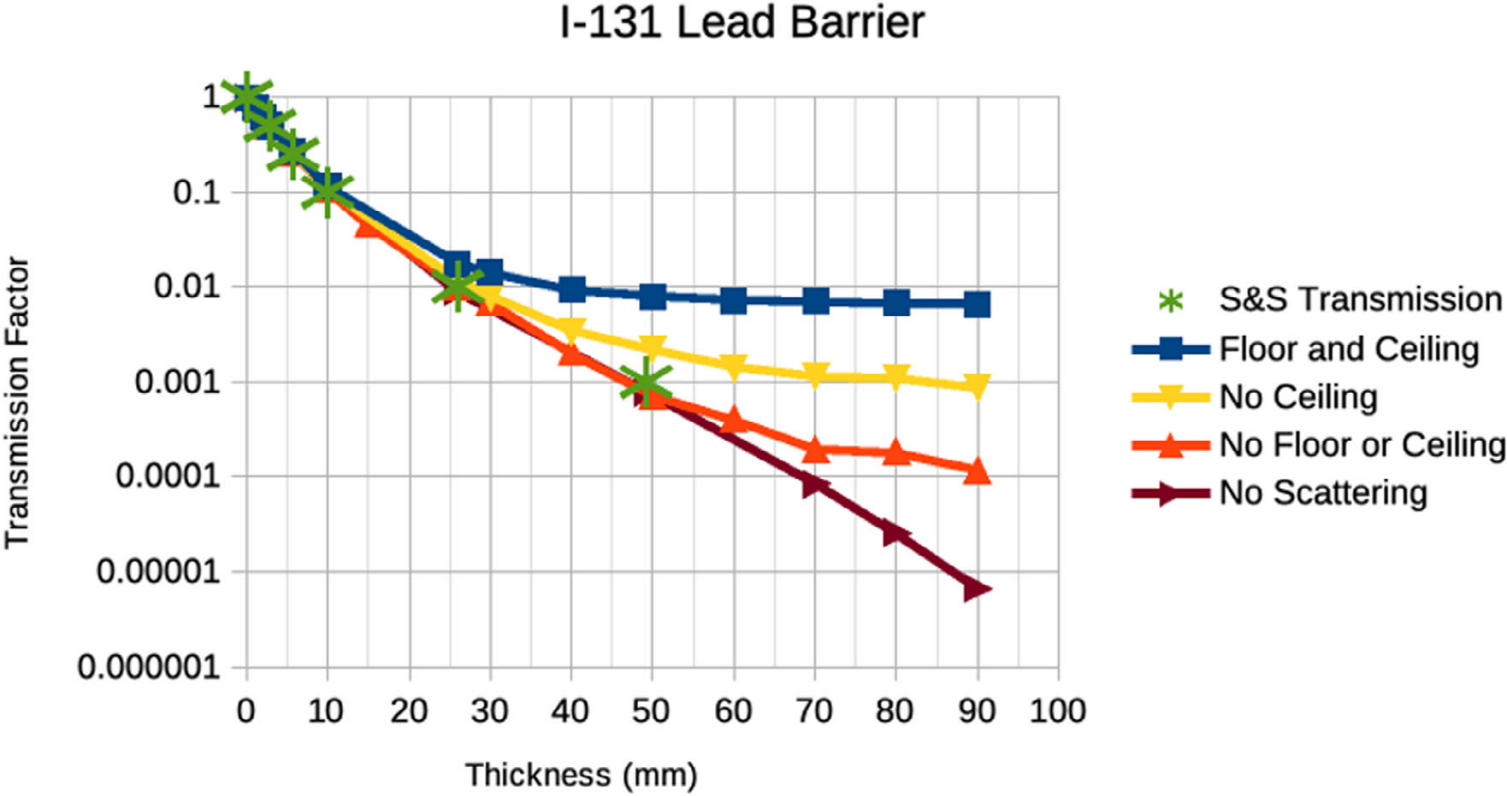
Broad beam transmissions curves for I‐131 and lead with and without scatter from ceiling and floor. Note that “no scattering” refers to a scenario with a barrier that extends to the edges of the simulation.

**FIGURE 10:**
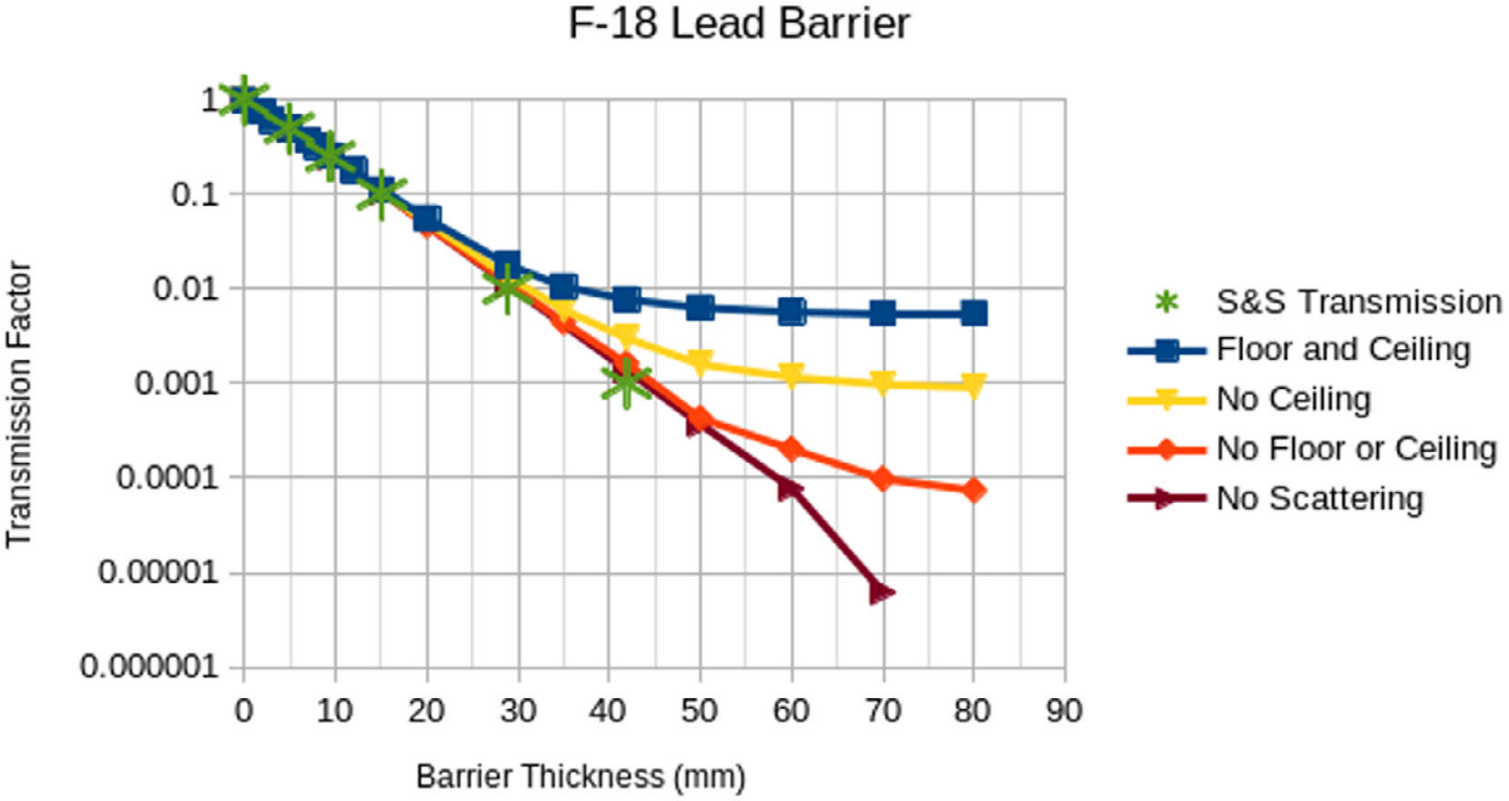
Broad beam transmissions curves for F‐18 and lead with and without scatter from ceiling and floor. Note that “no scattering” refers to a scenario with a barrier that extends to the edges of the simulation.

**FIGURE 11:**
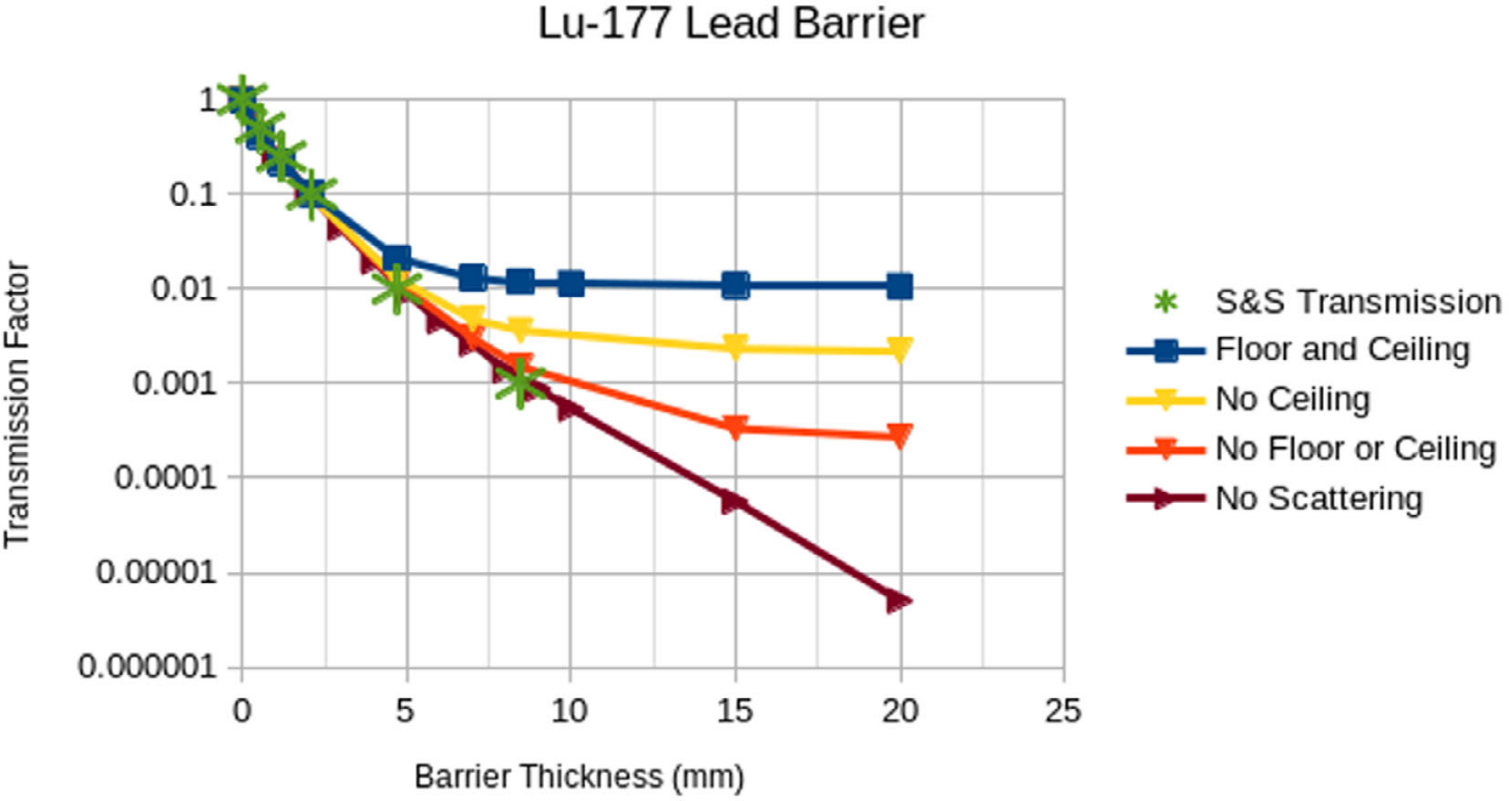
Broad beam transmissions curves for Lu‐177 and lead with and without scatter from ceiling and floor. Note that “no scattering” refers to a scenario with a barrier that extends to the edges of the simulation.

**FIGURE 12:**
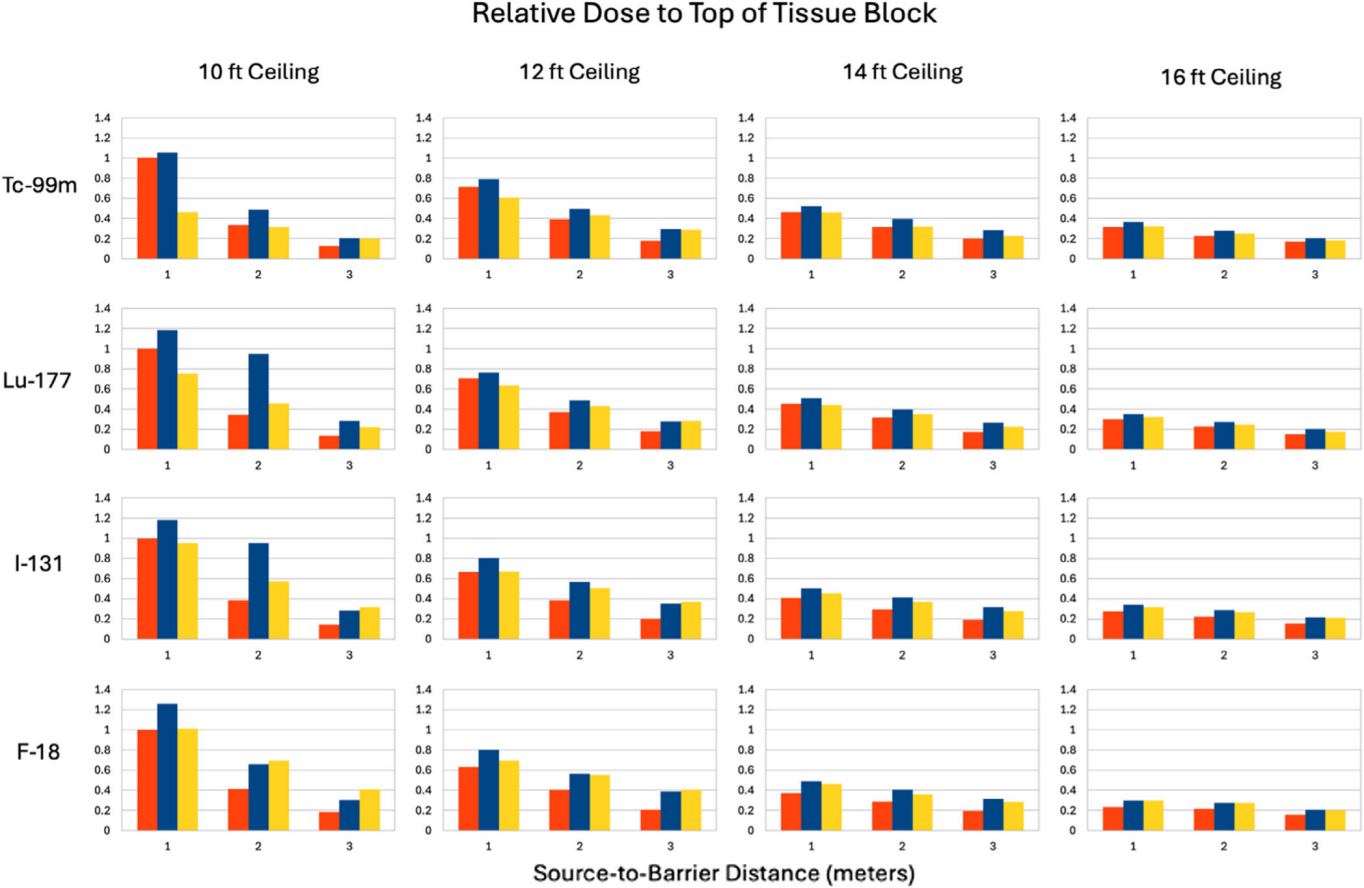
Summary of relative scatter onto the top of the tissue block for Tc‐99m, Lu‐177, I‐131, and F‐18 and four different ceiling heights of 3.05, 3.66, 4.27, and 4.88 m (10, 12, 14, and 16 feet). The distances on the horizontal axes are those between the central plane of the tissue block and the center of the barrier. The vertical axes are relative doses. The first (red), second (blue), and third (yellow) column of each triplet corresponds to the source distances of 1, 2, and 3 m from the barrier.

The plots in Figures 8, 9, 10, 11, 12 illustrate that the effect of scatter from the ceiling and floor is insignificant until reaching a transmission factor through the barrier of less than about 0.1 but then increases in relative contribution to the total dose as the barrier transmission factor decreases to about 0.01, below which the scattered contribution dominates that through the barrier such that further increasing of the barrier thickness is futile. The “S&S” data points in these plots refer to the fractional value layers reported by Smith and Stabin.^13^ Their work considered only photon emissions of a radionuclide that exceed 15 keV in energy and 100 ppm in abundance and applied buildup factors that are based upon two different methods in the literature to the theoretical narrow beam attenuation of lead.

**FIGURE 13:**
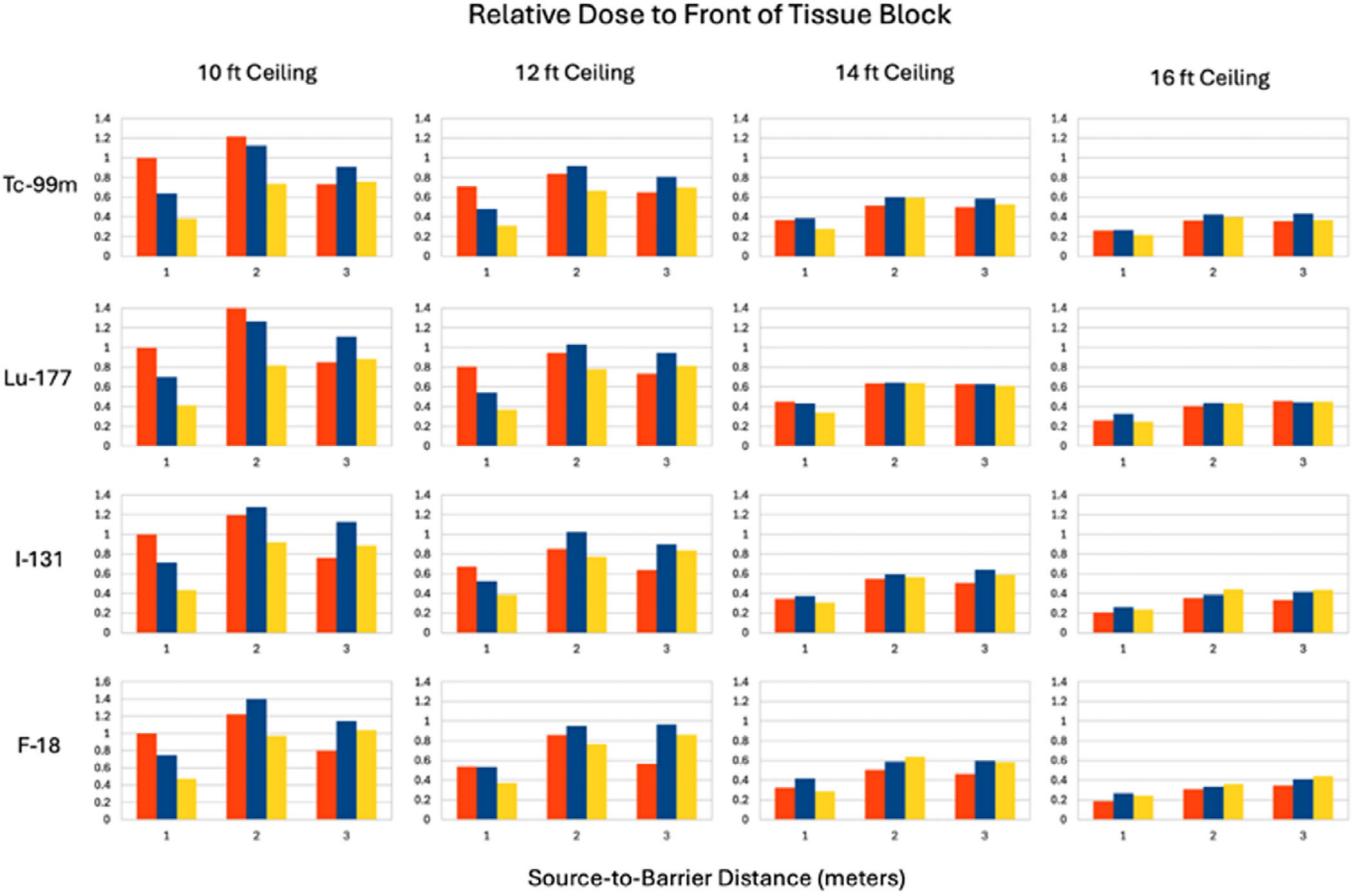
Summary of relative scatter onto the proximal face of the tissue block for Tc‐99m, Lu‐177, I‐131, and F‐18 and four different ceiling heights of 3.05, 3.66, 4.27, and 4.88 m (10, 12, 14 and 16 feet). The distances on the horizontal axes are those between the central plane of the tissue block and the center of the barrier. The vertical axes are relative doses. The first (red), second (blue), and third (yellow) column of each triplet corresponds to the source distances of 1, 2, and 3 m from the barrier.

The results are shown as bar charts for a given source radionuclide and distance from the barrier. The barrier was so thick that there was essentially no penetration directly through it. The vertical axes in Figures 12 and 13 are the normalized doses to the applicable ROIs.

All ceiling scatter simulations were run twice in GATE, which allows for estimating statistical uncertainty. The average percentage coefficient of variation (CV) of dose measurements in GATE was 7.7% for Tc‐99m, 9.6% for Lu‐177, 9.3% for I‐131, and 8.4% for F‐18 across all ceiling heights.

## 4 DISCUSSION

### 4.1 Example calculation for Lu‐177 treatment room

**FIGURE 14:**
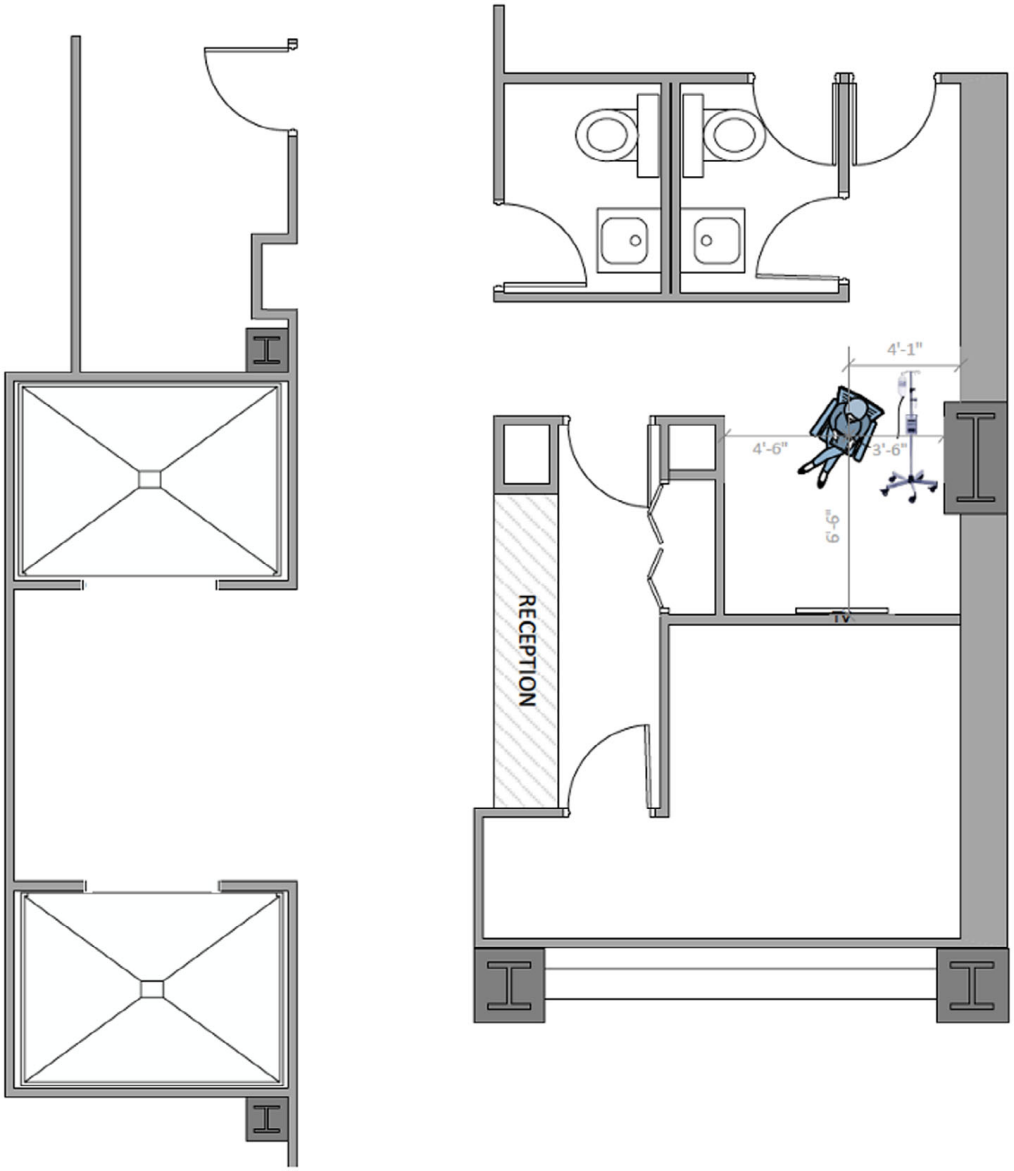
A nuclear medicine shielding scenario where a patient is undergoing radionuclide therapy in a temporarily repurposed waiting room.

Consider the sample room layout that is, shown in Figure 14 and calculate the amount of lead that would be needed in the wall that separates the patient from the office to the plan south.

The patient is modeled as a point source that is located 1.98 m (6′ 6″) from the wall. The wall itself is 11.4 cm (4.5″) thick and we wish to shield a point that is, 30 cm (1 foot) from the wall to the public dose limit in the United States of 20 µGy (20 µSv) a week based upon 1 mGy (1 mSv) a year. The total separation between the source and the point of concern is 2.40 m.

Assume that five patients a week are administered the typical 200 mCi (7.4 GBq) dosage of Lu‐177 DOTATATE and that each patient remains in the treatment room for 4 h. Thus, the weekly workload in the room is 4000 mCi‐hr ignoring the decay of the Lu‐177. From Table 4, the exposure rate constant of Lu‐177 is 0.181 R/mCi‐h at 1 cm and the f‐factor is 0.957 cGy/R. The weekly dose rate at the point of concern will be

$$\begin{array}{ccc} D & = & \frac{4000\mathrm{mCi}-\mathrm{hr}}{\mathrm{week}}\times \frac{0.181R}{\mathrm{mCi}-\mathrm{hr}}\times 9.57\times 10^{-3}\frac{\mathrm{Gy}}{R}\\ & & \times \;\frac{1}{{\left(240\;\mathrm{cm}\right)}^{2}}=120.3\mu \mathrm{Gy}/\mathrm{week} \end{array}$$

**TABLE 4:** Physical properties of radionuclides evaluated in this report.^12^, ^13^, ^18^

Nuclide	Half‐life^12^	Decay mode	Gamma constant at 1 cm (R/mCi‐h)^13^	f‐factor (cGy/R)^13^	Dominant photon energies (keV)^18^	HVL of lead (mm)^13^	TVL of lead (mm)^13^
Tc‐99m	6.02 h	Isomeric transition γ emission 89.07%^18^	0.795	0.959	140 (89.07%)	0.234	0.905
I‐131	8.02 days	$\beta^{-}$	2.2	0.963	364 (81.2%), 637 (7.26%)	2.74	9.93
Lu‐177	6.65 days	$\beta^{-}$	0.181	0.957	113 (6%), 208 (11%)	0.542	2.11
F‐18	109.8 min	$\beta^{+}$, EC	5.7	0.876	511 (194%)	4.95	15.1

Abbreviations: HVL, half‐value layer; TVL, tenth‐value‐layer.

A transmission factor of T = 20/120.3 = 0.166 is required in order to achieve the public dose limit at the point of concern. The thickness of lead that is required can be calculated using Equation 4 and the parameters of the Archer equation for Lu‐177 and lead that are given in Table 2:

$$\begin{array}{ccc} x & = & \left(\frac{1}{0.3855*0.2822}\right)\;\ln\left(\frac{0.166^{-0.2822}+\frac{1.071}{0.2822}}{1+\frac{1.071}{0.2822}}\right)\\ & = & 1.48\;\mathrm{mm} \end{array}$$

In some situations in which people are not continuously present in an area, an occupancy factor that is smaller than unity can be justified. Table 5 is adapted from NCRP 147 based upon the consensus of the authors.

**TABLE 5:** Recommended occupancy factors in nuclear medicine departments (modified from NCRP Report No. 147).

Area	Recommended Occupancy Factor
Offices, labs, pharmacies, receptionist areas, attended waiting rooms, kids’ play areas, imaging rooms, film reading areas, nursing stations, control rooms	1
Patient exam rooms, patient treatment rooms, nuclear medicine hot labs, PET uptake rooms	1/2
Corridors, patient rooms, employee lounges, staff rest rooms	1/5
Corridor doors	1/8
Public toilets, vending areas, storage rooms, outdoor areas with seating, unattended waiting rooms, patient holding	1/20
Outdoors, unattended parking lots, attics, stairways, unattended elevators, janitor's closets	1/40

Abbreviations:NCRP, National Council on Radiation Protection and Measurement; PET, positron emission tomography.

### 4.2 What should be protected?

This report has concentrated on the protection of human beings. However, the instruments that are used in nuclear medicine can be quite sensitive to ambient radiation levels and might also require protection. These devices include dose calibrators, well counters, and uncollimated gamma cameras. Although the results of this investigation indicate that scattering from the floor and ceiling is insignificant for the protection of people in most scenarios, it should be considered when designing rooms in which large activities could be stored for decay or where several therapeutic patients could be treated in close proximity to such instruments. It might be necessary in such situations to install barriers that extend all of the way from the floor to the deck of the floor above in order to reduce scattering in the air above the barrier.

### 4.3 Limitations

One limitation of this study is that it is purely computational and consequently is highly idealized and simplified. For example, it is highly unlikely that all nuclear medicine departments are going to have ceilings and floors made only of 3 in. (7.62 cm) of lightweight concrete. The materials are likely to be considerably more variable, and we have not explored the effects of that variability. Experimental measurements obtained in the field would help significantly with this but have not yet been conducted.

Another limitation of this study is that all radioactive sources have been modeled as point sources in air without accounting for the patient's self‐shielding except for the exclusion of emitted photons with energies below 15 keV. We primarily made this choice to allow for direct comparisons with experimental measurements. For example, the Joint Commission now requires that radiation protection surveys be performed to verify that the prescribed shielding has been properly installed. If the simulations included more patient‐like self‐attenuated sources, these radiation protection surveys would not be directly comparable.

We have found that different fitting programs sometimes give different parameters for the Archer equation even though the plots of the fits are visually equivalent. It is also noteworthy that the photon spectra of some radionuclides are different than the continuous radiographic x‐ray spectra that Archer originally modeled^15^.

### 4.4 Ceiling/floor scatter

The results in Figures 8, 9, 10, 11 suggest that scattering from floors and ceilings is real, but that it is not a major concern in most clinical situations, which do not require transmission factors smaller than 0.01. If/when consideration of ceiling scatter is warranted, Figures 12, 13 indicate that dose from scatter to the top falls off when the source and target are farther from the barrier, dose from scatter to the front is more apt to increase when the source and target are farther from the barrier, there is a greater scattering dose when the ceiling is lower (perhaps only because the path length is shorter for lower ceilings than for higher ceilings), and that on the proximal face of the tissue block the dose is higher when the source is farther than 1 m from the barrier. This implies that when the dose from scattering is a concern, the positions of the source and the target matter.

Considering the Lu‐177 treatment room sample calculations above, the required transmission factor is 0.166 and the required lead thickness is 1.48 mm, which according to Figure 11, results in negligible scatter off the ceiling or floor. However, if we consider an extreme hypothetical scenario where the time‐activity product is 18,000 mCi‐h/week (300 mCi constantly present for 60 h per week) and the distance is only 73.2 cm, then the required transmission factor would be 0.0034 and the required lead thickness would be 7.6 mm (as calculated above). In this extreme hypothetical scenario, Figure 11 indicates this shielding would be inadequate due to scatter off the ceiling and floor. This scenario would require installing a barrier from the top of the primary barrier up to the ceiling, which need not be so thick as the primary barrier because of the lower flux and the oblique angle of the rays that would intersect the upper barrier.

### 4.5 Nomenclature

Note that we discourage the use of the term “skyshine” in the context of scattering from the floor and the ceiling as, “skyshine” usually implies scatter off air and Figures 8, 9, 10, 11 imply that the scattering in air is at least an order of magnitude less significant than that from the floor and the ceiling in nuclear medicine.

## 5 SUMMARY AND CONCLUSIONS

In this report, we have used Monte Carlo simulations to provide broad beam transmission factors for Lu‐177, Tc‐99m, I‐131, and F‐18 in practical clinical scenarios. We have also specifically evaluated the effect of scatter off ceilings and floors. Buildup and scatter off ceilings and floors are insignificant when shielding for the first 90 percent of transmission (i.e., for transmission factors that are greater than 0.1), but these factors severely limit the ability to shield for transmission below 1% (i.e., for transmission factors that are less than 0.01). Despite the relatively low exposure rate constant of Lu‐177 compared to the other commonly used radionuclides in nuclear medicine, some degree of shielding is likely to be necessary for departments that are performing relatively high volumes of Lu‐177 therapies such as Lu‐177 DOTATATE and Lu‐177 vipivotide tetraxetan PSMA. Shielding for Lu‐177 (as with all radiopharmaceuticals) involves the consideration of broad radiation beams and thus requires a thorough characterization of radiation buildup.

## AUTHOR CONTRIBUTION

All authors conceived of the presented idea. Richard Wendt and Michael Oumano developed the theory and performed the experiments + computations. Richard Wendt and Michael Oumano prepared the manuscript. All authors discussed the results, reviewed the manuscript, and contributed to the final manuscript.

## CONFLICT OF INTEREST STATEMENT

The authors declare no conflicts of interest.
